# Nanoparticle-Based Drug Delivery in Cancer Therapy and Its Role in Overcoming Drug Resistance

**DOI:** 10.3389/fmolb.2020.00193

**Published:** 2020-08-20

**Authors:** Yihan Yao, Yunxiang Zhou, Lihong Liu, Yanyan Xu, Qiang Chen, Yali Wang, Shijie Wu, Yongchuan Deng, Jianmin Zhang, Anwen Shao

**Affiliations:** ^1^Department of Surgical Oncology, The Second Affiliated Hospital, School of Medicine, Zhejiang University, Hangzhou, China; ^2^Department of Radiation Oncology, The Second Affiliated Hospital, School of Medicine, Zhejiang University, Hangzhou, China; ^3^School of Pharmacy, Nanjing Medical University, Nanjing, China; ^4^Key Laboratory of Targeted Intervention of Cardiovascular Disease, Collaborative Innovation Center for Cardiovascular Disease Translational Medicine, Nanjing Medical University, Nanjing, China; ^5^Cancer Institute (Key Laboratory of Cancer Prevention and Intervention, China National Ministry of Education), The Second Affiliated Hospital, Zhejiang University School of Medicine, Hangzhou, China; ^6^Department of Neurosurgery, The Second Affiliated Hospital, Zhejiang University School of Medicine, Hangzhou, China

**Keywords:** nanoparticle, drug delivery, hybrid nanoparticles, targeted cancer therapy, drug resistance

## Abstract

Nanotechnology has been extensively studied and exploited for cancer treatment as nanoparticles can play a significant role as a drug delivery system. Compared to conventional drugs, nanoparticle-based drug delivery has specific advantages, such as improved stability and biocompatibility, enhanced permeability and retention effect, and precise targeting. The application and development of hybrid nanoparticles, which incorporates the combined properties of different nanoparticles, has led this type of drug-carrier system to the next level. In addition, nanoparticle-based drug delivery systems have been shown to play a role in overcoming cancer-related drug resistance. The mechanisms of cancer drug resistance include overexpression of drug efflux transporters, defective apoptotic pathways, and hypoxic environment. Nanoparticles targeting these mechanisms can lead to an improvement in the reversal of multidrug resistance. Furthermore, as more tumor drug resistance mechanisms are revealed, nanoparticles are increasingly being developed to target these mechanisms. Moreover, scientists have recently started to investigate the role of nanoparticles in immunotherapy, which plays a more important role in cancer treatment. In this review, we discuss the roles of nanoparticles and hybrid nanoparticles for drug delivery in chemotherapy, targeted therapy, and immunotherapy and describe the targeting mechanism of nanoparticle-based drug delivery as well as its function on reversing drug resistance.

## Introduction

Finding new and innovative treatments for cancer is a major problem across the world (Siegel et al., 2020). With an increase in the number of methods that can treat cancer and the concept of an individualized treatment, the therapeutic efficacy of some malignant tumors has greatly improved. Chemotherapy is a conventional and widely used cancer treatment method. While chemotherapy works through a number of different mechanisms, its major function includes indiscriminately killing vigorously growing cells, including tumor and normal cells, which causes some serious side effects including bone marrow suppression, hair loss, and gastrointestinal reactions (Zitvogel et al., 2008). Therefore, developing drugs that more accurately target tumor cells, instead of normal cells, has been the purpose of a large proportion of cancer-related research in the past few decades. Although the emergence of targeted therapy has made great progress in precision therapy, there are still many unavoidable adverse effects, and the development of drug resistance has always been a problem. Currently, cancer remains the second leading cause of death, and current therapies for many cancers are inadequate. Hence, increasingly more studies are seeking precise therapy of cancer and solutions for drug resistance.

Over the last few decades, nanotechnology has been increasingly used in medicine, including applications for diagnosis, treatment, and tumor targeting in a safer and more effective manner. Nanoparticle (NP)-based drug delivery systems have shown many advantages in cancer treatment, such as good pharmacokinetics, precise targeting of tumor cells, reduction of side effects, and drug resistance (Dadwal et al., 2018; Palazzolo et al., 2018). NPs used in drug delivery systems are usually designed or chosen based on their size and characteristics according to the pathophysiology of the tumors. Mechanically, nano-carriers in cancer therapy target to tumor cells through the carrier effect of NPs and the positioning effect of the targeting substance after being absorbed. Next, they release the drugs to tumor cells in order to induce killing. Drugs located on the inside of the nano-carriers include traditional chemotherapy agents and nucleic acids, indicating that they can play a role in both cytotoxic and gene therapy (Chen et al., 2015). In addition, for some poorly soluble drugs, NPs offer a platform that can help encapsulate them and deliver the drugs into circulation (Kipp, 2004; Zhang et al., 2008). Due to the size and surface characteristics of NPs and their function of enhancing permeability and retention, nano-carriers can increase the half-life of drugs and induce their accumulation into tumor tissues (Bertrand et al., 2014; Kalyane et al., 2019). Meanwhile, the targeting system protects normal cells from the cytotoxicity of drugs, which helps ease the adverse effects of cancer therapy. For example, doxorubicin-loaded PEGylated liposomes reduced cardiotoxicity compared to free doxorubicin (O’Brien et al., 2004). Additionally, nanoparticle albumin-bound paclitaxel exhibited less side effects and allowed higher tolerated doses than solvent-based taxanes (Cortes and Saura, 2010). In addition to chemotherapy and gene therapy, various studies have reported the application of NP drugs in immunotherapy and ablation treatment for cancer (Riley and Day, 2017; Yoon et al., 2018). The nanoparticle-based drug delivery system is believed to enhance immunotherapy, as well as reverse the tumor immunosuppressive microenvironment (Zang et al., 2017).

In recent years, an increasing number of nanotherapeutic drugs have been commercialized or entered the clinical stage. The first phase I clinical trial that used a targeted nanoparticle-based system to deliver small interfering RNA (siRNA) in patients with solid cancers was conducted in 2010 (Davis et al., 2010). Another clinical study reported a more favorable tumor treatment efficacy of an actively targeted polymeric nanoparticle containing the chemotherapeutic docetaxel (DTXL) compared to a solvent-based DTXL formulation (Hrkach et al., 2012). The development of hybrid NPs has made even more progress in the arena of NP-based drug delivery systems. Hybrid NPs combine the properties of different NPs, thereby enhancing the function and stability of each drug delivery system (Mottaghitalab et al., 2019). In addition, NPs have shown certain advantages when it comes to anti-tumor multidrug resistance (MDR), as they provide platforms for drug combination therapy as well as inhibit the function of some mechanisms of drug resistance, such as efflux transporters on cell membranes (Li et al., 2016). Nowadays, nanoparticle-based therapy has been reported to have potential in overcoming MDR in several types of cancers, including breast cancer (Alimoradi et al., 2018), ovarian cancer (Wang et al., 2018b), and prostate cancer (Zhang J. et al., 2019). Nanotechnology in medicine has opened a new stage of cancer treatment, and the combination of these two fields deserves more in-depth research. This review outlines the basic principles of the application of the nano-carrier system in cancer therapy, presents the current challenges, and describes the directions of future research.

## NPs in Cancer Therapy

**FIGURE 1:**
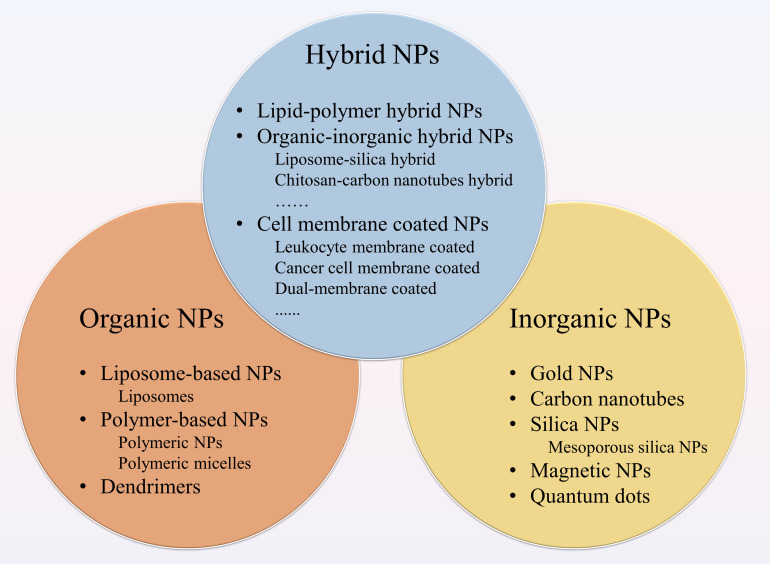
Different types of nanoparticles (NPs) for cancer therapy. NPs applied to drug delivery systems include organic NPs, inorganic NPs and hybrid NPs. The organic NPs contain liposome-based NPs, polymer-based NPs and dendrimers. Among polymer-based NPs, polymeric NPs and polymeric micelles are common. The inorganic NPs consist of gold NPs (Au NPs), carbon nanotubes, silica NPs, magnetic NPs, and quantum dots. Hybrid NPs combine the advantages of different NPs, including lipid-polymer hybrid NPs, organic-inorganic hybrid NPs, and cell membrane-coated NPs.

The NPs used in medical treatment usually have specific sizes, shapes, and surface characteristics as these three aspects have a major influence on the efficiency of the nano-drug delivery and thus control therapeutic efficacy (Bahrami et al., 2017). NPs with a diameter range of 10 to 100 nm are generally considered suitable for cancer therapy, as they can effectively deliver drugs and achieve enhanced permeability and retention (EPR) effect. Smaller particles can easily leak from the normal vasculature (less than 1–2 nm) to damage normal cells and can be easily filtered by kidneys (less than 10 nm in diameter) (Venturoli and Rippe, 2005), while particles that are larger than 100 nm are likely to be cleared from circulation by phagocytes (Decuzzi et al., 2009). Moreover, the surface characteristics of NPs can influence their bioavailability and half-life. For instance, NPs that are coated with hydrophilic materials such as polyethylene glycol (PEG) lessen the opsonization and therefore avoid clearance by the immune system (Yang et al., 2014). Therefore, NPs are generally modified to become hydrophilic, which increases the time period of drugs in circulation and enhances their penetration and accumulation in tumors (Perrault et al., 2009; Wong et al., 2011; Yang et al., 2014). Collectively, the various characteristics of NPs determine their therapeutic effect in cancer management. Different types of NPs for cancer therapy are shown in Figure 1 and the following text will describe their respective advantages in tumor treatment.

## Organic NPs

Organic NPs have been widely explored for decades and contain many types of materials. Liposome, the first nano-scale drug approved for clinical application (Zylberberg and Matosevic, 2016), consists of an outer lipid layer and a core entrapping either hydrophobic or hydrophilic drug. Liposomes can carry out many functions by modifying the lipid layer structure, including imitating the biophysical characteristics (e.g., mobility and deformation) of living cells (Hua and Wu, 2013; Lemière et al., 2015), which can help achieve the purpose of more effective therapeutic drug delivery. With decades of research, the development of liposomes has gone through several generations. With regard to cancer therapy, liposomes provide a good platform for *in vivo* delivery of many anti-tumor drugs, such as doxorubicin and paclitaxel, among other chemotherapeutic agents, as well as nucleic acids (Chen et al., 2010; Wang X. et al., 2017). In the field of breast and prostate cancer (Yari et al., 2019), the application of liposomes has been increasingly common (Satsangi et al., 2015; Tang B. et al., 2020). Multiple paclitaxel liposomes have been demonstrated to have higher anti-tumor efficiency and improved bioavailability compared to free paclitaxel (Han et al., 2020). Liposomal doxorubicin has been proven to reduce cardiotoxicity and has comparable efficacy in breast cancer (O’Brien et al., 2004; Geisberg and Sawyer, 2010). Furthermore, liposome-based nanosystems have also offered an option for drug combination, which can enhance the therapeutic effect (Eloy et al., 2016; Chen et al., 2017) and even reverse the drug resistance (Meng et al., 2016). Nowadays, more varieties of liposome-based drugs have entered into clinical use for cancer treatment (Misra et al., 2010).

Polymer-based NPs are another type of NP with specific structural arrangements for drug delivery formed by different monomers (Amreddy et al., 2018). Polylactic-co-glycolic acid (PLGA), a common polymeric NP, encompasses co-polymerization of glycolic acid and lactic acid. Given its better biocompatibility and biodegradation, as well as the EPR effect, PLGA is widely used as a carrier for drug delivery (Acharya and Sahoo, 2011; Saneja et al., 2019). Additionally, dendrimers are another class of polymers that have been applied to nanomedicine. They are versatile and biocompatible macromolecules that are characterized by a three-dimensional branch structure (Nanjwade et al., 2009; Sherje et al., 2018). Their multiple functional groups on the surface enhance the capability of loading and delivering therapeutic agents. Furthermore, polymeric micelles, which are characterized by polymer self-assembly into nano-aggregates as they are composed of amphiphilic copolymers, constitute another kind of widely investigated polymer NPs (Zhou et al., 2018). The hydrophobic core enables the insoluble anticancer drugs to be absorbed and delivered smoothly, while the hydrophilic segment increases stability, thus reducing the uptake of the drug by the reticuloendothelial system and prolonging their time period in circulation (Cagel et al., 2017).

## Inorganic NPs

Inorganic NPs have the advantages of a higher surface area to volume ratio. They have a wide and easily modified surface conjugation chemistry and facile preparation, although this usually occurs at the expense of poorer biocompatibility and biodegradability (Jiang et al., 2016). The inorganic NPs that have been studied include gold NPs (AuNPs), carbon nanotubes (CNTs), quantum dots, magnetic NPs (MNPs), and silica NPs (SNPs). AuNPs are the most widely studied inorganic NPs, and mixed monolayer-protected clusters based on the gold core are considered to be a promising candidate in the drug delivery system (Han et al., 2007). The gold core is inert and non-toxic, and surface-functionalized AuNPs have been proven to enhance drug accumulation in tumors as well as to overcome the drug resistance (Cheng et al., 2013). Moreover, AuNPs are thought to be involved in multimodal cancer treatment including gene therapy, photothermal therapy and immunotherapy (Han et al., 2007; Jiang et al., 2016; Riley and Day, 2017).

carbon nanotubes are a type of tubular material that have been shown to have broad potential in the drug delivery field due to their unique biological, physical, and chemical properties. As a result, they have been used to deliver anticancer agents including doxorubicin, paclitaxel, and methotrexate siRNA for a variety of cancers (Madani et al., 2011). Meanwhile, CNTs produce heat when they are exposed to near-infrared radiation, which could be applied to thermal ablation for cancer therapy (Luo et al., 2013).

Mesoporous silica nanoparticle carriers are a type of SNPs which are suitable for drug delivery (Almeida et al., 2014; Gao et al., 2019). The large internal pore volume enables them to encapsulate the maximum amount of anticancer drugs, and the supramolecular components act as a cap, allowing capture and release of drugs (Cheng et al., 2019; Lei et al., 2019). Due to better pharmacokinetics and treatment efficacy, as well as high stability, SNPs are considered one of the best vehicles for drug delivery (Zhang F. et al., 2017; Xu et al., 2019). Moreover, porous silicon NPs have shown great potential in immunotherapy as its immunoadjuvant properties include promotion of antigen cross presentation, polarization of lymphocytes and secretion of interferon-γ (IFN-γ) (Fontana et al., 2017b).

Magnetic NPs (MNPs) used for drug delivery usually contain metal or metal oxide NPs. In order to improve the stability and biocompatibility, MNPs are commonly coated with organic materials, including polymers and fatty acids. They have been shown to demonstrate high efficacy in chemotherapy and gene therapy for cancer treatment (Basoglu et al., 2018; Mandriota et al., 2019). Furthermore, magnetic hyperthermia using MNPs can achieve thermal ablation of tumors, which offers alternative cancer treatment (Hoopes et al., 2017; Legge et al., 2019).

## Hybrid NPs

As both organic and inorganic NPs have their own advantages and disadvantages, combining the two in a single hybrid drug delivery system endows the multifunctional carrier with better biological properties that can enhance treatment efficacy as well as reduce drug resistance (Mottaghitalab et al., 2019).

Lipid-polymer hybrid NPs, which consist of an inner polymeric core and a lipid shell, have been demonstrated to be a promising drug delivery platform in the treatment of pancreatic cancer (Hu et al., 2010; Zhao et al., 2015), breast cancer (Gao et al., 2017; Li et al., 2019), and metastatic prostate cancer (Wang Q. et al., 2017). This type of hybrid NPs combines the high biocompatibility of lipids with the structural integrity provided by polymer NPs, and are therefore capable of encapsulating both hydrophilic and hydrophobic drugs in order to achieve a better therapeutic effect (Cheow and Hadinoto, 2011; Zhang R.X. et al., 2017). Meanwhile, this system can be effectively internalized by cancer cells (Su et al., 2013) and avoids fast clearance by the reticuloendothelial system (Hu et al., 2015).

The combination of organic and inorganic hybrid nano-materials is a common method of NP design. For example, a liposome-silica hybrid (LSH) nanoparticle consists of a silica core and a surrounding lipid bilayer and has been synthesized and shown to be valid in delivering drugs to kill prostate and breast cancer cells (Colapicchioni et al., 2015). The LSH nanoparticle has also been reported to offer a platform for the synergistic delivery of gemcitabine and paclitaxel to pancreatic cancer in a mouse model of the disease (Meng et al., 2015). Kong et al. (2015) created an advanced nano-in-micro platform by assembling the porous silicon NPs and giant liposomes onto a microfluidic chip, and co-delivery of synthesized DNA nanostructures and drugs in this platform was proven to significantly enhance cell death of doxorubicin-resistant breast cancer cells. Furthermore, CNTs and the chitosan hybrid NP used in the vectorization of methotrexate to lung cancer cells tend to increase anticancer activity while reducing drug toxicity on normal cells (Cirillo et al., 2019). Moreover, half-shells of metal multilayers (such as manganese and gold) and PLGA hybrid NPs have the potential of combining targeted drug delivery and hyperthermia, which can enhance the destruction of tumor cells (Park et al., 2009).

The hybridization of natural biomaterial with organic or inorganic NPs is another method for NP design. For example, cell membrane coating nanotechnology is emerging and has increasingly gained more attention. This technology tends to bestow the NPs with biological characteristics directly by coating NPs with naturally derived cell membranes, which enhances the potency and safety of conventional NPs (Fang et al., 2018). The coatings include cell membranes derived from leukocytes, red blood cells, platelets, cancer cells, and even bacteria. Parodi et al. (2013) have shown that coating nanoporous silicon particles with a cell membrane which is purified from leukocytes can prevent the nano-carrier from clearance by phagocytes, and the characteristics of this hybrid particle allow the drug to have extended time period in circulation, leading to increased accumulation in the tumor. Similarly, some studies have utilized cancer cell membrane-cloaked mesoporous silica NPs for cancer treatment, which improves the stability and targeting ability of nano-carriers (Liu et al., 2019). Moreover, the development of dual-membrane coated NPs can further enhance the function of NPs. For instance, erythrocyte-platelet hybrid and erythrocyte-cancer hybrid membrane-coated NPs were proven to exhibit better stability and longer circulation life (Dehaini et al., 2017; Wang et al., 2018a; Jiang et al., 2019).

Furthermore, (Wong et al., 2011) proposed a multistage NP delivery system to achieve deep penetration into tumors by changing the size and characteristics of NPs at different stages. In their study, the size change of NPs was achieved by protease degradation of the cores of 100-nm gelatin NPs within the tumor microenvironment in order to release 10-nm quantum dot NPs.

## Mechanisms of Targeting

**FIGURE 2:**
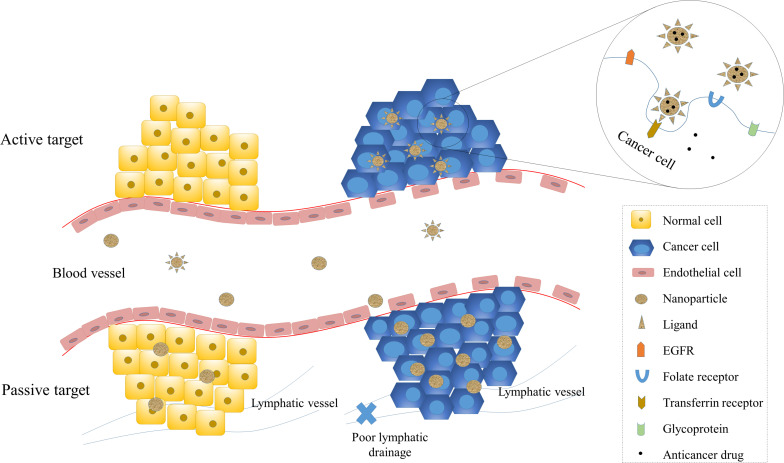
Passive and active targeting of NPs to cancer cells. Targeting of NPs enhance therapeutic efficiency and reduce systemic toxicity. Passive targeting of NPs is mainly achieved by the enhanced permeability and retention (EPR) effect, which exploits the increased vascular permeability and weakened lymphatic drainage of cancer cells and enables NPs to target cancer cells passively. Active targeting is achieved by the interaction between ligands and receptors. The receptors on cancer cells include transferrin receptors, folate receptors, glycoprotein (such as lectin), and epidermal growth factor receptor (EGFR).

Targeting of cancer cells specifically is a vital characteristic of nano-carriers for drug delivery, as it enhances the therapeutic efficacy while protecting normal cells from cytotoxicity. Numerous studies have been carried out to explore the targeting design of NP-based drugs. In order to better address the challenges of tumor targeting and the nano-carrier system design, it is crucial to first understand tumor biology and the interaction between nano-carriers and tumor cells. The targeting mechanisms can be broadly divided into two categories, passive targeting and active targeting (Figure 2).

## Passive Targeting

Passive targeting is designed to utilize the different characteristics of tumor and normal tissue. In passive targeting, the drugs are successfully delivered to the target site in order to play a therapeutic role. High proliferation of cancer cells induces neovascularization, and large pores in the vascular wall lead to a worsening permselectivity of tumor vessels compared to normal vessels (Carmeliet and Jain, 2000). The rapid and defective angiogenesis enables macromolecules, including NPs, to leak from blood vessels that supply the tumor and accumulate within tumor tissue. Meanwhile, the poor lymphatic drainage associated with cancer increases the retention of NPs, allowing the nano-carriers to release their contents to tumor cells. These processes cause the EPR effect, one of the driving forces of passive targeting (Maeda, 2001). The EPR effect is influenced by the size of NPs, as many studies have demonstrated that smaller NPs have better penetrability but do not leak into normal vessels (Torchilin, 2005; Carita et al., 2018). On the other hand, larger particles are more likely to be cleared by the immune system (Sykes et al., 2014).

In addition to the EPR effect, the tumor microenvironment is also an important factor in the passive delivery of nanomedicines. Glycolysis is one of the metabolic characteristics of cancer cells and is the main source of energy for cancer cell proliferation (Pelicano et al., 2006). Glycolysis yields an acidic environment and reduces the pH of the tumor microenvironment. Subsequently, some pH-sensitive NPs are triggered by the low pH level and are able to release drugs within the vicinity of cancer cells (Lim et al., 2018).

However, there are some limitations with regards to passive targeting, including non-specific drug distribution, non-universal existence of the EPR effect and different permeability of blood vessels across various tumors (Jain, 1994).

## Active Targeting

Active targeting specifically targets cancer cells through direct interactions between ligands and receptors. The ligands on the surface of NPs are selected to target the molecules that are overexpressed on the surface of cancer cells, which allows them to distinguish targeted cells from healthy cells (Shi et al., 2011; Kamaly et al., 2012). The interaction between ligands on NPs and the receptors on the surface of cancer cells induces receptor-mediated endocytosis, which allows internalized NPs to successfully release therapeutic drugs (Farokhzad and Langer, 2009). Therefore, active targeting is particularly suitable for macromolecular drug delivery, such as proteins and siRNAs. The types of targeting moieties include monoclonal antibodies, peptides, amino acids, vitamins, and carbohydrates (Danhier et al., 2010). These ligands specifically bind to receptors on targeted cells, and the widely investigated receptors include transferrin receptor, folate receptor, glycoproteins, and the epidermal growth factor receptor (EGFR).

### Targeting to Cancer Cells

Transferrin, a type of serum glycoprotein, functions to transport iron into cells. Transferrin receptors are overexpressed in most solid tumor cells and are expressed at low levels in normal cells. Thus, transferrin-conjugated NPs are used as an active targeting method to deliver drugs for cancer treatment (Amreddy et al., 2015; Liu et al., 2015; Santi et al., 2017). Compared to unmodified NPs, transferrin-modified NPs have been shown to exhibit higher cellular uptake efficiency and enhanced intracellular delivery of drugs (Cui et al., 2017). Moreover, evidence indicates that transferrin-conjugated polymeric NPs play a significant role in overcoming drug-resistant chemotherapy (Soe et al., 2019).

Folic acid, a type of vitamin, is essential in nucleotide synthesis. It is internalized by a folate receptor that is expressed on few normal cell types. However, the alpha isoform of folate receptor (FR-α) is overexpressed in approximately 40% of human cancers, while FR-β is expressed on the surface of hematopoietic cancers (Low and Kularatne, 2009). Thus, the folate receptor-targeting strategy by folate-conjugated nanomaterials has been widely used for cancer treatments (Muralidharan et al., 2016; Samadian et al., 2016).

In addition, cancer cells usually express various types of glycoproteins, including lectins, which are non-immunological proteins that recognize and specifically bind to certain carbohydrates (Minko, 2004). Targeting cancer cell-surface carbohydrates by lectins conjugated to NPs constitutes the direct lectin targeting pathway, while inversely targeting lectins on cancer cells using carbohydrates moieties that are incorporated into NPs is referred to as the reverse lectin targeting pathway (Minko, 2004; Obaid et al., 2015).

Epidermal growth factor receptor is a member of the ErbB family of tyrosine kinase receptors. EGFR, which is overexpressed in varieties of cancers, is involved in several processes of tumor growth and progression and has already been utilized as a target for cancer treatment (Nicholson et al., 2001; Sigismund et al., 2018). For example, targeting human epidermal receptor-2 (HER-2) is a common therapy for HER-2 positive breast and gastric cancer. Hence, NPs that have been designed to incorporate modified ligands that bind to EGFR in order to target EGFR-overexpressed cancer cells is a promising method of drug delivery (Alexis et al., 2008). Furthermore, conjugating two cancer-specific ligands into a single NP is another way of active targeting, as it can help improve target specificity (Balasubramanian et al., 2014).

### Targeting to Endothelium

Some NPs do not directly target cancer cells but instead have an effect on angiogenesis, which is another method of cancer treatment. The interaction between vascular endothelial growth factor (VEGF) and VEGF receptors (VEGFRs) plays an essential role in vascularization (Apte et al., 2019). Additionally, targeting VEGFR-2 and VEGFR-3, two major VEGF receptors, simultaneously by liposomes has been shown to enhance therapeutic efficacy (Orleth et al., 2016).

Integrins are cell surface receptors for extracellular matrix proteins that play an important role in tumor cell migration and invasion (Desgrosellier and Cheresh, 2010). The αvβ3 integrin is highly expressed in tumor neovascular endothelial cells, rather than the resting endothelial and normal cells, and is important in the calcium-dependent pathway that induces endothelial cell migration (Nisato et al., 2003). Hood et al. have reported the favorable treatment efficacy of cationic NPs coupled with an αvβ3 integrin-targeting ligand for gene delivery into tumor-bearing mice (Hood et al., 2002). In addition, αvβ3 integrin is associated with VEGFR-2 signaling (Ruoslahti, 2002), and blocking αvβ3 integrin-binding can lead to a reduction in VEGF signaling, indicating that targeting αvβ3 integrin can enhance the effectiveness of anti-VEGFR treatment.

Vascular cell adhesion molecule-1 (VCAM-1) is an immunoglobulin-like glycoprotein that is also expressed on the surface of the tumor endothelium and is involved in angiogenesis by interacting with vascular endothelial cells. Overexpression of VCAM-1 can be observed in various cancers (Dienst et al., 2005), indicating its potential role in the active targeting of NPs for drug delivery. A study by Pan et al. (2013) have reported the high efficiency of VCAM-1 targeted NPs in a breast cancer model.

Moreover, matrix metalloproteinase (MMP), a component of the tumor microenvironment, is engaged in extracellular matrix remodeling and tumor neovascularization (Lia et al., 2009). MMP-sensitive NPs have been reported to play a potential antitumor effect in several types of cancers, including breast cancer, pancreatic cancer, and melanoma (Mansour et al., 2003; Xiao et al., 2018; Cun et al., 2019).

## Mechanisms of NPs in Overcoming Drug Resistance

Drug resistance is still a major problem in cancer treatment, despite the fact that methods of cancer therapy are increasing. Multidrug resistance leads to a failure of various types of cancer treatments, leading to cancer progression and poor prognosis. The mechanisms of tumor drug resistance include cellular and physiological factors, such as overexpression of ATP binding cassette (ABC) transporters (e.g., efflux transporter) (Litman et al., 2000), defective apoptotic machineries, interstitial fluid pressure, and acidic and hypoxic tumor microenvironment. Nanotechnology applied to drug delivery for cancer treatment has been shown to play a significant role in overcoming drug resistance (Table 1).

**TABLE 1:** The application of nanoparticle-based drug delivery system for overcoming drug resistance.

**Targeted pathway**	**Mechanisms (in addition to antitumor efficiency)**	**Drugs**	**References**
Efflux transporters	Bypass efflux transporters	NP itself	(Murakami et al., 2011)
	Inhibit efflux transporters	COX-2 inhibitors	(Zhang S. et al., 2019)
		P-gp-targeted siRNA	(Patil et al., 2010; Navarro et al., 2012)
		miRNA-495	(He et al., 2019)

Apoptosis	Inhibit anti-apoptosis pathway	Bcl-2-targeted siRNA	(Wang et al., 2006; Saad et al., 2008; Chen et al., 2009; Choi et al., 2019)
		NF-κB inhibitors (pyrrolidine dithiocarbamate/curcumin)	(Fan et al., 2010; Misra and Sahoo, 2011; Zhao et al., 2019)
	Activate pro-apoptosis pathway	Ceramide	(Devalapally et al., 2007; van Vlerken et al., 2010)
		p53 gene therapy	(Prabha and Labhasetwar, 2004; Choi et al., 2008)

Efflux transporters and apoptosis	Inhibit efflux transporter expression meanwhile promoting apoptosis through down-regulation of Bcl-2 and NF-κB expression	Bcl-2 convertor gene-loaded NPs	(Cheng et al., 2018)
		Resveratrol	(Zhao et al., 2016; Singh et al., 2018)
	Inhibit efflux transporters and promote apoptosis by inducing mitochondrial outer membrane permeabilization	Mitochondria-targeted NPs	(Wang et al., 2020)

Hypoxia	Silence the HIF-1α gene	HIF-1α siRNA	(Zhao et al., 2015; Luan et al., 2018; Hajizadeh et al., 2020)
	Inhibit the function of HIF-1α	HIF-1α inhibitors	(Reddy et al., 2011)
	Indirectly downregulate HIF-1α expression	Inhibitors of the PI3K/Akt/mtor pathway	(Zhang et al., 2018)
		HSP90 inhibitors	(Long et al., 2018)

*Abbreviations: Bcl-2 = B cell lymphoma-2; COX-2 = cyclooxygenase 2; HIF-1α = hypoxia-inducible factor 1α; HSP90 = heat shock protein 90; NF-κB = nuclear factor kappa B; NPs = nanoparticles; P-gp = P-glycoprotein; PI3K = phosphoinositol-3-kinase; siRNA = small interfering RNA.*

## Targeting Efflux Transporters

Efflux transporters belong to a family of ABC transporters that have been proven to play essential roles in drug resistance. Efflux transporters reduce intercellular drug concentration by pumping the drug out of the cell, leading to a failure of treatment. Among them, P-glycoprotein (P-gp), one of the most widely investigated efflux transporters, is overexpressed in several drug-resistant tumors (Schneider and Hunke, 1998; Allen et al., 2000). In addition, high expression of P-gp has been associated with poor treatment-response in many tumors, such as breast (Chintamani et al., 2005) and ovarian cancer (Agarwal and Kaye, 2003). A myriad of studies have demonstrated that some chemotherapeutics-loaded NPs can bypass the exposure of anti-tumor drugs to efflux transporters, since NPs largely enter the cell through endocytosis instead of diffusion and release the drug at a perinuclear site within the cell, away from cell membranes and efflux pumps (Murakami et al., 2011). The nanoparticle-based drug delivery system can modify the control of drug release. For example, several researches have utilized low pH level and redox as triggers for drug release in NPs (Yu et al., 2018; Kundu et al., 2019). Furthermore, NPs, such as polymers, also act as MDR modulators (Qin et al., 2018). For instance, micelles based on amphiphilic diblock polymer of N-(2-hydroxypropyl) methacrylamide (HPMA) and poly (propylene oxide) block (PPO) are able to inhibit P-gp (Braunová et al., 2017).

Combination therapy is another strategy to treat drug-resistant cancers. To this end, NP-based combination therapy has been able to overcome the problem of pharmacokinetic differences between different drugs by assembling multiple therapeutic agents within a single drug carrier, thereby fighting drug resistance and improving the therapeutic effect of cancer therapy (Cuvier et al., 1992; Schneider and Hunke, 1998; Allen et al., 2000; Susa et al., 2009). In addition to bypassing efflux transporters, inhibiting their expression and function would be another option to cope with efflux transporter-mediated drug resistance. This strategy can be achieved by designing NPs that encapsulate both efflux pump inhibitors and chemotherapeutics (Soma et al., 2000), or by reducing the ATP that is supplied to the efflux pump (Wang et al., 2018c). As COX-2 has been shown to be involved in P-gp-mediated multidrug resistance in cancer, a selective COX-2 inhibitor can down-regulate the P-gp expression (Sui et al., 2011). Indeed, a recent study conducted by Zhang S. et al. (2019) confirmed that co-delivery of COX-2 inhibitors and doxorubicin by NPs reversed the multidrug resistance of breast cancer cells. Furthermore, several studies have revealed that co-delivery of P-gp-targeted siRNA and anticancer drugs by NPs helps overcome drug-resistant cancers, which is exerted through inhibiting the expression of ABC transporters (Patil et al., 2010; Navarro et al., 2012). A recent study revealed the effectiveness of overcoming drug resistance in lung cancer therapy by combining miRNA-495 and doxorubicin into a cancer cell membrane-coated silica nanoparticle, results of which indicated that miR-495 effectively down-regulated P-gp expression in multidrug-resistant cancer cells (He et al., 2019).

In addition, (Bai et al., 2013) reported that nanoparticle-mediated drug delivery to the tumor neovasculature was able to overcome P-gp-expressing multidrug resistant cancer by targeting KDR receptors, which are highly expressed in the tumor vasculature. This system showed a more effective anti-tumor function when compared to chemotherapeutic and P-gp inhibitor combination therapy.

## Targeting Apoptotic Pathway

Defective apoptotic machineries enable cancer cells to evade apoptosis and increase survival, thereby contributing to drug resistance in cancer (Viktorsson et al., 2005). The defective apoptotic pathway is often triggered by deregulating Bcl-2 and nuclear factor kappa B (NF-κB). Bcl-2 is a widely investigated anti-apoptotic protein, is highly expressed in many cancers, and is a key player in drug resistance, suggesting its potential as a target for reversing drug resistance. Accumulating evidence has indicated that co-delivery of Bcl-2-targeted siRNA and chemotherapeutics by NPs is an alternative to overcoming drug resistance in cancer (Wang et al., 2006; Saad et al., 2008; Chen et al., 2009; Choi et al., 2019). Moreover, NF-κB inhibitors have been used in NP-based combination therapy, including pyrrolidine dithiocarbamate (PDTC) (Fan et al., 2010) and curcumin (Misra and Sahoo, 2011; Zhao et al., 2019).

In addition to suppressing anti-apoptotic moieties, the activation of pro-apoptotic compounds can also be used to combat apoptotic pathway-mediated drug resistance. For example, combining ceramide with the chemotherapeutic drug paclitaxel augments the therapeutic efficacy of various drug-resistant tumor models (Devalapally et al., 2007; van Vlerken et al., 2010). On the other hand, a recent study revealed that ceramide is able to restore the expression of wild-type p53 protein, an important tumor suppressor, by modulating alternative pre-mRNA splicing. In this process, NPs offers a more effective platform to deliver ceramide into cancer cells that carry p53 missense mutations, an important cancer phenomenon (Khiste et al., 2020). As p53 plays a significant role in apoptosis, reinstating p53 function or other tumor suppressors is considered a potential way to overcome drug resistance in cancer. Therefore, p53 gene therapy utilizing a nanoparticle-based delivery system has been further researched. Transfecting the p53 gene by cationic solid lipid NPs and PLGA has been reported in lung (Choi et al., 2008) and breast cancer cells (Prabha and Labhasetwar, 2004), respectively. These results show the effective induction of apoptosis and inhibition of tumor growth.

Furthermore, some NP-based drug delivery systems function by inhibiting efflux pumps, as well as promoting apoptosis. Cheng et al. (2018) utilized an amphiphilic cationic NP complex encapsulating paclitaxel and the Bcl-2 convertor gene in order to inhibit drug-resistant liver cancer cell growth. Findings from the study showed that this NP complex impaired P-gp-induced drug efflux and the activation of apoptosis. This work is a pioneer study that was able to successfully overcome both pump- and non-pump-mediated drug resistance. In addition, co-delivery of doxorubicin and resveratrol in NPs have shown significant cytotoxicity on doxorubicin-resistant breast cancer cells by inducing apoptosis through the down-regulation of Bcl-2 and NF-κB expression, as well as through the inhibition of efflux transporter expression (Zhao et al., 2016). Similarly, another study demonstrated the effectiveness of folic acid-conjugated planetary ball-milled NPs that were encapsulated with resveratrol and docetaxel for the treatment of multidrug-resistant prostate cancer. Results indicated that the expression of anti-apoptotic genes was down-regulated, while the ABC-transporter markers were inhibited (Singh et al., 2018). Moreover, mitochondria-targeted NPs also showed an effect on both efflux transporters and apoptotic pathway. Targeting to mitochondria led to a reduction in ATP production, which is required by ABC transporters. Additionally, paclitaxel-loaded TPP-Pluronic F127-hyaluronic acid nanomicelles caused mitochondrial outer membrane permeabilization (MOMP), which resulted in the release of cytochrome C and activation of caspase-3 and caspase-9, leading to apoptosis of drug-resistant lung cancer cells (Wang et al., 2020).

## Targeting Hypoxia

Hypoxia is another factor that contributes to multidrug resistance (Jing et al., 2019). Due to irregular blood vessels, as well as the increased oxygen demand of rapidly proliferating cancer cells, some cancer cells are often in a hypoxic state. Hypoxia induces the drug resistance of tumors in many ways. For instance, slowly dividing cells in hypoxic regions can escape from cytotoxic chemotherapeutics such as alkylating agents and antibiotics. Additionally, hypoxia produces a gradient of oxygen within the tumor, thereby increasing tumor heterogeneity and promoting a more aggressive phenotype. Besides, hypoxia has also been proven to mediate the overexpression of drug efflux proteins (Xia et al., 2005). During the process, hypoxia-inducible factor 1α (HIF-1α) plays an essential role, and overexpression of HIF-1α has been observed in many human cancers (Vadde et al., 2017). Therefore, targeting HIF-1α is another treatment method for overcoming drug resistance (Rey et al., 2017).

There is also an extensive study on the application of NPs in the treatment of hypoxia. Silencing the HIF-1α gene is one of the ways to inhibit hypoxic environment. Several studies have reported the effectiveness of nanosystems containing HIF-1α siRNA to overcome drug resistance in cancer (Zhao et al., 2015; Luan et al., 2018; Hajizadeh et al., 2020). HIF-1α inhibitors have also shown therapeutic efficacy in reducing hypoxia-mediated drug resistance (Reddy et al., 2011). In addition to directly inhibiting the function of HIF-1, indirect inhibition of HIF-1 signaling has also been previously considered. For example, the PI3K/Akt/mTOR pathway can regulate the expression of HIF-1α, and the inhibition of this pathway down-regulates HIF-1α expression, thereby enhancing the sensitivity of MDR cells to cancer treatment (Zhang et al., 2018). In this process, NPs, such as PLGA-PEG (Tang X. et al., 2020) and PEGylated and non-PEGylated liposomes (Iwase and Maitani, 2012), can offer better platforms to achieve combination therapy. In addition, heat shock protein 90 (HSP90) is required for HIF-1 transcriptional activity, and inhibition of HSP90 can also down-regulate HIF-1α expression (Semenza, 2007). The HSP90 inhibitor in 17AAG-loaded NPs has been shown to dramatically improve bladder cancer treatment (Long et al., 2018).

## The Role of NPs in Cancer Immunotherapy

The development of immunotherapy has brought cancer treatment into a new era. NPs not only play an important role in delivery chemotherapy but have also shown great potential for applications in immunotherapy. Cancer immunotherapy is mainly achieved by activating the anti-tumor immune response (Zang et al., 2017). NP-associated immunotherapy includes nanovaccines, artificial antigen-presenting cells (aAPCs), and targeting of the immunosuppressed tumor microenvironment (TME) (Zang et al., 2017).

Nanovaccines deliver tumor-associated antigens (TAAs) and adjuvants to APCs, such as dendritic cells (DCs) (Paulis et al., 2013). Additionally, NPs can be used as adjuvants themselves to increase APC antigen presentation and promote DC maturation, leading to the activation of the anti-tumor function of cytotoxic T cells (Shao et al., 2015; Yang et al., 2018). NPs, such as liposomes, gold NPs, PLGA NPs, micelles, and dendrimers all have the capability of cytoplasmic delivery of TAAs into DCs, thus enhancing the immune response against tumor cells (Guo et al., 2015). Among different types of NPs, inorganic NPs such as mesoporous silica and polymers such as acetylated dextran (AcDEX) have been shown to function as an adjuvant in immunotherapy, leading to a stimulation of the immune response (Fontana et al., 2017a, b). Unlike nanovaccines, artificial APCs function with MHC-antigen complexes and co-stimulatory molecules that directly bind to T cell receptors (TCRs) and co-stimulatory receptors on T cells, respectively, resulting in T cell activation (Perica et al., 2014). Targeting the immunosuppressive TME is mainly achieved by targeting tumor-associated macrophages (TAMs), myeloid derived suppressor cells (MDSCs), and regulatory T cells (Tregs), all of which are important cell types in the TME (Shao et al., 2015). Furthermore, in order to minimize interactions with the reticuloendothelial system, NPs are usually modified with PEG (Zang et al., 2017).

In addition, the combination of chemotherapy and immunotherapy is a promising strategy of cancer treatment. For example, one study showed that co-loading of the chemotherapeutic agent Nutlin-3a and the cytokine GM-CSF in spermine-modified AcDEX NPs led to improved proliferation of cytotoxic CD8(+) T cells and stimulated immune response, leading to tumor cell death while avoiding toxicity in immune cells (Bauleth-Ramos et al., 2017). Alternative approaches of combined chemo-immunotherapy includes co-delivery of chemotherapeutics and monoclonal antibodies into porous silicon NPs, which have been effective in stimulating complement activation, antibody-dependent cell cytotoxicity (ADCC), and immune response against cancer cells (Li et al., 2018).

## Conclusion and Future Perspectives

Nanotechnology applied to cancer therapy has led to a new era of cancer treatment. Various types of NPs, including organic and inorganic NPs, have already been widely used in the clinical treatment of several cancer types. Compared to traditional drugs, NP-based drug delivery systems are associated with improved pharmacokinetics, biocompatibility, tumor targeting, and stability, while simultaneously playing a significant role in reducing systemic toxicity and overcoming drug resistance. These advantages enable NP-based drugs to be widely applied to chemotherapy, targeted therapy, radiotherapy, hyperthermia, and gene therapy. Moreover, nanocarrier delivery systems provide improved platforms for combination therapy, which helps overcome mechanisms of drug resistance, including efflux transporter overexpression, defective apoptotic pathway, and hypoxia tumor microenvironment. According to different mechanisms of MDR, NPs that are loaded with varieties of targeting agents combined with cytotoxic agents can achieve the reversal of drug resistance.

With increasing research, various types of hybrid NPs have shown improved properties for delivery and aroused more attention. Further studies on the biological characteristics of individual cancers will lead to more precise research directions for these drugs. Furthermore, designing hybrid NPs that are more suitable for cancer therapy and engineering NPs that target cancer cells more specifically using targeting moieties merits further exploration. Notably, the interactions between NPs and the immune system are complex (Najafi-Hajivar et al., 2016). The NP size, shape, composition, and surface are all the factors that affect the interactions of NPs with the immune system. Although nanovaccines and artificial APCs have demonstrated increased efficacy compared to traditional immunotherapy, the clinical efficacy of this treatment remains unsatisfactory, and the safety and tolerance of these new approaches need to be further investigated. Moreover, developing immunomodulatory factor-loaded NPs may improve the effectiveness of vaccines for immunotherapy. Accordingly, a better understanding of the TME and a further investigation of the crosstalk between NP-based drug delivery systems and tumor immunity are warranted for drug design and exploitation.

## Author Contributions

YY, YZ, AS, and SW conceptualized the research project. YY, LL, YX, and YW drafted the manuscript. YZ, AS, and QC reviewed and modified the manuscript. AS and YD supervised the research and led the discussion. All authors approved the final version of the manuscript.

## Conflict of Interest

The authors declare that the research was conducted in the absence of any commercial or financial relationships that could be construed as a potential conflict of interest.

## Abbreviations

17AAG: 17-allylamino-17-demethoxygeldanamycin
ABC: ATP binding cassette
ADCC: antibody dependent cell cytotoxicity
APC: antigen-presenting cell
AuNPs: gold nanoparticles
Bcl-2: B cell lymphoma-2
CNT: carbon nanotubes
COX-2: cyclooxygenase 2
DC: dendritic cell
DTXL: docetaxel
EGFR: epidermal growth factor receptor
EPR: enhanced permeability and retention
FR- α: alpha isoform of folate receptor
GM-CSF: granulocyte-macrophage colony-stimulating factor
HER-2: human epidermal receptor-2
HIF-1 α: hypoxia-inducible factor 1 α
HSP90: heat shock protein 90
IFN- γ: interferon γ
KDR: kinase-insert domain-containing receptor
LSH: liposome-silica hybrid
MDCS: myeloid derived suppressor cell
MDR: multidrug resistance
MMP: matrix metalloproteinase
MNPs: magnetic nanoparticles
MOMP: mitochondrial outer membrane permeabilization
mTOR: mammalian target of rapamycin
NF- κ B: nuclear factor kappa B
NPs: nanoparticles
PDTC: pyrrolidine dithiocarbamate
PEG: polyethylene glycol
P-gp: P-glycoprotein
PI3K: phosphoinositol-3-kinase
PLGA: polylactic-co-glycolic acid
SNP: silica nanoparticles
TAA: tumor-associated antigen
TAM: tumor-associated macrophage
TCR: T cell receptor
TME: tumor microenvironment
Tregs: regulatory T cells
VCAM-1: vascular cell adhesion molecule-1
VEGF: vascular endothelial growth factor
VEGFRs: VEGF receptors.
